# Identification of candidate biomarkers correlated with poor prognosis of breast cancer based on bioinformatics analysis

**DOI:** 10.1080/21655979.2021.1960775

**Published:** 2021-08-12

**Authors:** Gang Chen, Mingwei Yu, Jianqiao Cao, Huishan Zhao, Yuanping Dai, Yizi Cong, Guangdong Qiao

**Affiliations:** a Department of Breast Surgery, The Affiliated Yantai Yuhuangding Hospital of Qingdao University, Yantai, Shandong, P.R. China; b Department of Orthopedics, The Affiliated Yantai Yuhuangding Hospital of Qingdao University, Yantai, Shandong, P.R. China; c Reproductive Medicine Centre, The Affiliated Yantai Yuhuangding Hospital of Qingdao University, Yantai, Shandong, P.R. China; d Department of Medical Genetics, Liuzhou Maternal and Child Health Hospital, Guangxi, P.R. China

**Keywords:** Bioinformatics, breast cancer, prognosis biomarker, gene expression omnibus, hub genes

## Abstract

Breast cancer (BC) is a malignancy with high incidence among women in the world. This study aims to screen key genes and potential prognostic biomarkers for BC using bioinformatics analysis. Total 58 normal tissues and 203 cancer tissues were collected from three Gene Expression Omnibus (GEO) gene expression profiles, and then the differential expressed genes (DEGs) were identified. Subsequently, the Gene Ontology (GO) function and Kyoto Encyclopedia of Genes and Genome (KEGG) pathway were analyzed to investigate the biological function of DEGs. Additionally, hub genes were screened by constructing a protein–protein interaction (PPI) network. Then, we explored the prognostic value and molecular mechanism of these hub genes using Kaplan–Meier (KM) curve and Gene Set Enrichment Analysis (GSEA). As a result, 42 up-regulated and 82 down-regulated DEGs were screened out from GEO datasets. The DEGs were mainly related to cell cycles and cell proliferation by GO and KEGG pathway analysis. Furthermore, 12 hub genes (*FN1, AURKA, CCNB1, BUB1B, PRC1, TPX2, NUSAP1, TOP2A, KIF20A, KIF2C, RRM2, ASPM*) with a high degree were identified initially, among which, 11 hub genes were significantly correlated with the prognosis of BC patients based on the Kaplan–Meier-plotter. GSEA reviewed that these hub genes correlated with KEGG_CELL_CYCLE and HALLMARK_P53_PATHWAY. In conclusion, this study identified 11 key genes as BC potential prognosis biomarkers on the basis of integrated bioinformatics analysis. This finding will improve our knowledge of the BC progress and mechanisms.

## Introductions

Breast cancer (BC) is the most common malignant tumor among women in the world and the leading cause to woman death[1], [2]. Alone United States up to 276,480 new women cases accounts for 30% of female cancers in the past years [3]. According to the molecular features of estrogen receptor (ER), progesterone receptor (PR), and human epidermal growth factor receptor 2 (Her2), BC could be separated into four subtypes. Namely, Luminal A (ER+/PR+, Her2–); LuminalB (ER+/PR+, Her2+); HER2+ (ER-/PR–, Her2+); and triple-negative breast cancer (TNBC) (ER-/PR–, Her2–) [4]. Based on the different molecular types, different measures will be carried out during clinical treatment [5]. Despite modern advances in target therapy method, the result of treating BC is still unsatisfactory because of drug resistance and recurrence. Thus, understanding the molecular mechanisms of BC and identifying novel potential prognostic biomarkers to improve the prognosis of BC are urgently needed.

Previous studies screened the biomarkers predictors and function enrichment, mostly using online tools [6,7], such as GEO2R, DAVID or KOBAS et al. Actually, the majority of the online database are not precise enough, due to the slow update. Besides, the online DE analysis tool GEO2R seems not to normalize original data, so there might be a great deviation for each probe.

In this study, we identified several key genes that could be used as sensitivity biomarkers for the diagnosis of BC based on Gene Expression Omnibus (GEO) database. We downloaded three different region source gene expression profiles (GSE29044, GSE42568, and GSE50428) from the GEO database consisting of 58 normal breast tissue samples and 203 BC tissue samples. Then, the limma package of R software and Venn diagram online tool were applied to differential expressed genes (DEGs) in the three datasets above. Furthermore, Gene Ontology (GO) function and Kyoto Encyclopedia of Genes and Genomes (KEGG) pathway analysis were conducted by the R software, and the newest annotation files were downloaded from the official website, respectively. We constructed a protein and protein interaction (PPI) network with Cytoscape and the hub genes were screened by the cytoHubba plugin. For the validation of the DEGs, the BC section in the database consisting of 1102 BC tissues and 113 normal tissues was downloaded using TCGA-Assembler [8] package of R software, and those DEGs from GEO were verified by the TCGA database. Finally, only 11 genes were selected as BC potential prognosis biomarkers by KM-plot, and GSEA analyses were involved to study the potential molecular mechanisms of these hub genes. To know our results reliability, we chose fewest reported non-hub genes and verified using BC patients sample, and it is consistent with our result In conclusion, our study provides some potential sensitive biomarkers for BC patients and promotes an understanding of the molecular mechanisms of BC progression.

## Materials and methods

### Data source and processing

The GEO (http://www.ncbi.nlm.nih.gov/geo/) database is a free public database of microarrays and is used for gene expression datasets and platform records [9]. The gene expression profiles of GSE29044, GSE42568, GSE50428 were chosen from the GEO database. GSE29044 was based on the GPL570 platform, containing 36 normal breast tissues and 73 BC tissues. GSE42568 was based on the GPL570 platform, containing 17 normal breast tissues and 104 BC tissues. GSE50428 was based on the GPL13648 platform, containing 5 normal breast tissues and 26 BC tissues. The downloaded data was studied using the Perl (Practical Extraction and Report Language, Version 5.30.2) software; then, log2 transformation and Z score standardization were performed on all data of gene expression.

### Identification of DEGs

DEGs between BC samples and normal breast samples were identified using the limma (version 3.30.0) package of R (version 3.5.1) software. The DEGs with FC≥ 1.5 or FC≤1/1.5 and adjust P < 0.05 were considered as the cutoff criteria. Then, we used Venn software online (http://bioinformatics.psb.ugent.be/webtools/Venn/) to obtain the common DEGs in all three independent cohorts.

### Functional enrichment analysis of DEGs

The GO annotation gives us a conspicuous meaningful for the variety of biological functional from microarray and other big datasets [10]. KEGG is a systematic gene and genomic function information database, which is stored in the PATHWAY segments [11]. GO and KEGG annotations were downloaded from the official website, respectively (http://current.geneontology.org/products/pages/downloads.html, https://www.genome.jp/kegg-bin/get_htext?hsa00001+3101, 2021–01-05 download), and enrichment of DEGs was performed using hypergeometric distribution formula of R software. We regarded P-value ≤ 0.05 with fold change more than 2 as a statistically significant difference and significant enrichment.

### PPI network construction and hub gene identification

After enrichment of the DEGs, we constructed the PPI network of all DEGs based on String online database (https://string-db.org/) [12] and Combined score greater than 0.9 as the cutoff criterion. The network was loaded to the Cytoscape (version 3.7.0) software, and cytoHubba plugin was carried out to predict hub genes [13].

### Validation of the hub genes in the TCGA database

The TCGA_BRCA dataset was downloaded using the TCGA-Assembler (version 2.0.6) package of R software [8] and then the up- and down-regulated hub gene expression levels were verified by the DESeq package of R software. Then, the potential function of the hub genes was analyzed by the GSEA (version 4.1.0).

### Breast cancer tissues

Seventeen breast cancer patients treated at the Department of Breast Surgery, The Affiliated Yantai Yuhuangding Hospital of Qingdao University were selected in our study. All the patients were signed informed consent forms approved by the Institutional Review Board of Yantai Yuhuangding Hospital.

### RNA isolation and Q-PCR

Total RNA was isolated from BC patients tissue samples using Trizol reagent as the manufacturer’s direction (Sparkjade, Qingdao, China). Following, 0.5 μg RNA from each sample was reversed to cDNA by the SPARK script II RT Plus Kit (Sparkjade, Qingdao, China). Then, Q-PCR was carried out using SYBR Green qPCR Mix kit (With ROX) (Sparkjade, Qingdao, China), following manufacturer’s instructions. Finally, the expression level of mRNA was calculated by the 2^−ΔΔCT^ formula.

### Survival analysis

To further investigate the value of hub genes in breast cancer patients, the Kaplan–Meier plotter (http://kmplot.com/analysis/) analysis was conducted [7]. BC database was applied to estimate the prognosis values of hub genes. If the p-value ≤ 0.05, it would be considered statistically significant.

### Statistical analysis

All statistical analyses were performed using R software. The data was expressed as the mean ± standard deviation (SD) from the dataset. Statistical analyses were conducted two-tailed and none paired Student’s t-test by the GraphPad Prism 5 (GraphPad Inc., San Diego, CA, USA) software, and p ≤ 0.05 were considered as statistical significance [14].

## Results

### Identification of DEGs in BC

**Figure 1.**
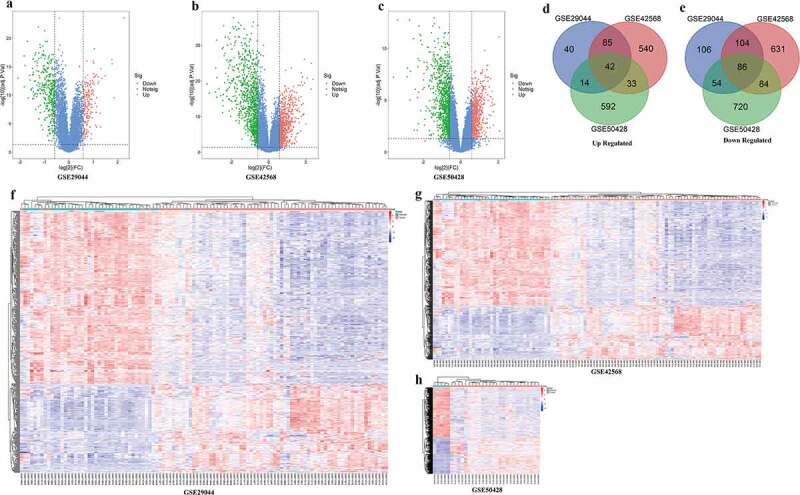
DEGs of three GEO profiles

Three gene expression profiles (GSE29044, GSE42568, and GSE50428) were selected in this study. Data from each GEO data set were respectively analyzed using limma package of R software to screen DEGs (FC ≥ 1.5 or FC≤1/1.5 and adjust P < 0.05). A total of 182, 705, 681 up-regulated and 351, 914, 944 down-regulated genes were filtered from GSE29044, GSE42568 and GSE50428, respectively. The volcano plot and heat map of DEGs were shown in (Figure 1(a-h). In addition, 41 and 86 overlapped up and down-regulssated DEGs were screened out by online Venn software from three gene expression profiles (Figure 1(d-e)).

(A-C, The volcano map of DEGs three GEO profiles. F-H, The heat map of DEGs three GEO profile. D, E, Venn diagram of three GEO profiles DEGs.)

### GO functional and KEGG pathway enrichment analysis of DEGs

**Figure 2.**
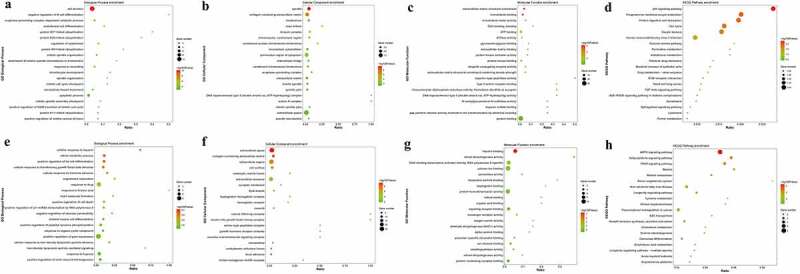
GO function and KEGG pathway analysis of DEGs

All DEGs enrichment score was calculated by the hypergeometric distribution of R software. For the up-regulated DEGs, Biological Process (BP) terms are most significantly enriched in cell division, negative regulation of B cell differentiation, and anaphase-promoting complex-dependent catabolic process. Cellular Component (CC) terms are most significantly enriched in spindle, collagen-containing extracellular matrix, and kinetochores. As for the Molecular Function (MF), extracellular matrix structural constituent, microtubule binding, and microtubule motor activity are mostly enriched (Figure 2(a-c)). Besides, for the down-regulated DEGs, BP terms are most significantly enriched in the cellular response to heparin, retinol metabolic process, and positive regulation of fat cell differentiation. CC terms are most significantly enriched in the extracellular space, collagen-containing extracellular matrix, and extracellular region. As for the MF, heparin binding, retinal dehydrogenase activity, and DNA-binding transcription activator activity, RNA polymerase II-specific are mostly enriched (Figure 2(e-g)). The method of enrichment is as same as that of the GO enrichment analysis. The up-regulated DEGs were most significantly enriched in the p53 signaling pathway, progesterone-mediated oocyte maturation, protein digestion and absorption. As for the down-regulated DGEs, they are mainly enriched in the AMPK signaling pathway, Adipocytokine signaling pathway, and PPAR signaling pathway (Figure 2(d-h)).

(A-D, GO function, and KEGG pathway analysis of up-regulated DEGs. E-H, GO function and KEGG pathway analysis of down-regulated DEGs.)

### PPI network construction and hub gene screening

**Figure 3.**
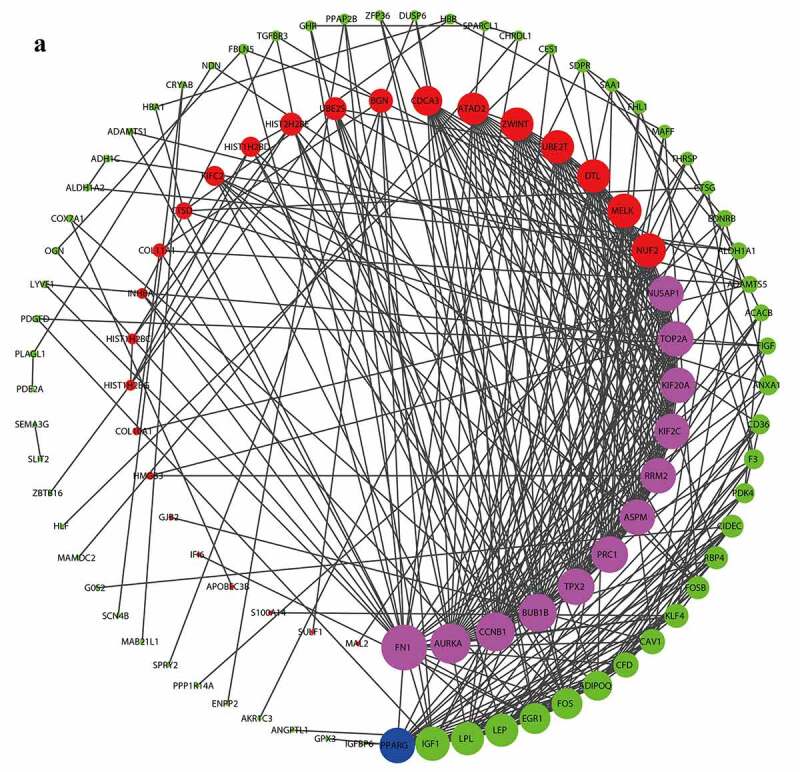
PPI analysis of hub genes of DEGs

The PPT network was constructed by the STRING online database and visualized by the Cytoscape software. The degree of the per node was calculated by the Cytohubba plugin, and the top 5 degrees of DEGs are considered as the hub genes of BC, 12 up-regulated (*FN1, AURKA, CCNB1, BUB1B, PRC1, TPX2, NUSAP1, TOP2A, KIF20A, KIF2C, RRM2, ASPM*) and 1 down-regulated (*PPARG*) (Figure 3).

(A, Red color is up-regulated DEGs, purple is up-regulated hub genes, green is down-regulated genes, blue is down-regulated hub genes.)

### DEGs validation

**Figure 4.**
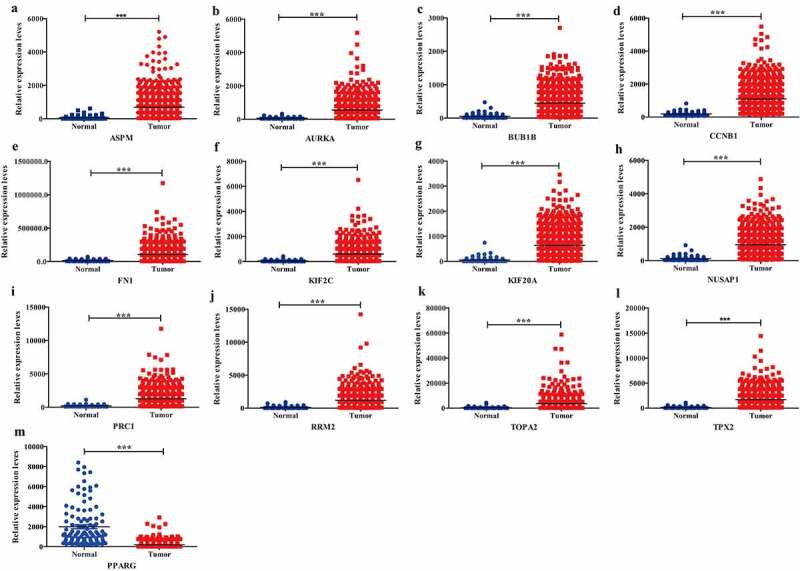
Expression validation of 13 hub targets in BC compared with adjacent tissues from TCGA data sets

To further investigate the 13 candidate hub genes, we validated their expression in the TCGA_BRCA dataset. The expression levels of all these 13 hub genes were consistent with the results of GEO profiles analysis (Figure 4).

(A-L, Up-regulated hub genes of BC from TCGA database. M, Down-regulated hub genes of BC from TCGA database.)

**Figure 5.**
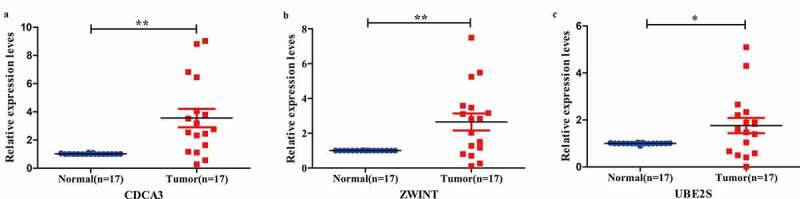
Non-hub genes validation in BC compared with adjacent tissues from patients

In the mean time, we chose least reported non-hub three genes (*CDC3A, ZWINT, and UBE2S*) and verified in BC samples by the Q-PCR. The expression levels of these three genes were consistent with GEO profiles (Figure 5).

(A-C. CDC3A and ZWINT, UBE2S relative expression levels, respectively.)

### Survival analysis of the identified hub genes

**Figure 6.**
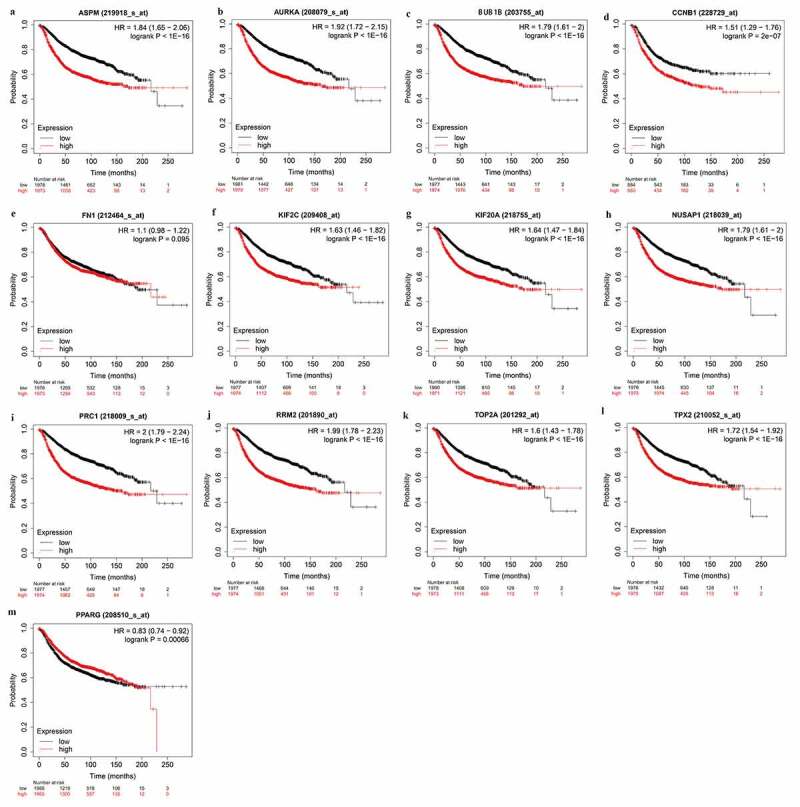
The prognostic gene signature of hub genes in the BC patients

To deeply clarify the role of hub genes in BC patients prognosis, the overall survival of hub genes was analyzed using KM-plot (http://kmplot.com/analysis/). The KM plot showed that all the hub genes had a significant difference between high and low expression levels, except *FN1* (Figure 6). This result indicated that the 12 hub genes have prognostic significance for BC patients.

(A-L, Overall survival of up-regulated hub genes. M, Overall survival of down-regulated hub genes.)

### Hub gene GSEA analysis

**Figure 7.**
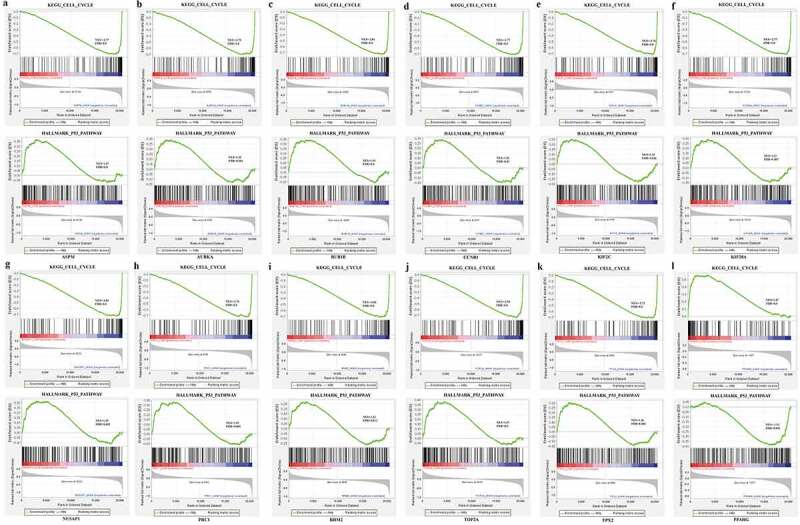
Enrichment plots from GSEA

To evaluate the potential mechanism of these hub genes in BC, GSEA was performed to get the underlying pathway from the GSEA dataset. The expression levels of these genes from TCGA_BRCA were separated into two parts using median. From the GSEA results, we could know that these hub genes were highly related to the ‘KEGG_CELL_CYCLE’ and ‘HALLMARK_P53_PATHWAY’ gene set (Figure 7). The result of GSEA indicated a significant difference (FDR ≤ 0.05) in the enrichment of MSigDB Collection and was consistent with GO and KEGG enrichment.

(A-K, Enrichment plots from GSEA of up-regulated hub genes. Enrichment plots from GSEA of down-regulated hub genes.)

## Discussion

As one of the most common cancers and the No.1 killer among women malignancies in the world, breast cancer brings women severe hurts physically and psychologically [15–17]. For the past ten years, the treatment of BC has many problems, such as drug resistance and poor prognosis, which reveals BC still is a high-risk disease. Therefore, exploring the molecular pathogenesis of BC is crucial to fully understand the development and prognosis of BC is imminent. Although there are a lot of bioinformatics paper reported about BC, they are almost based on the online tool, DAVID database with slow update rate. Obviously, some of the results are incomprehensive. In our study, we download newest annotation from official website GO and KEGG. Tidied and calculated enrichment using hypertension formula. The results would be more considerate.

In this study, in order to select effective biomarkers of BC and identify their potential molecular mechanisms, we studied three GEO profiles and found 124 overlapping DEGs, containing 42 up-regulated genes and 86 down-regulated genes. After all the analyses we performed, 12 BC target genes (*AURKA, CCNB1, BUB1B, PRC1, TPX2, NUSAP1, TOP2A, KIF20A, KIF2C, RRM2, ASPM, PPARG*) were identified. The detailed information of these genes is as follows.

From the GO function enrichment, we could know that *AURKA, PRC1, BUB1B, CCNB1, ASPM, and KIF2C* hold the same function of cell division. *AURKA* is a serine/threonine kinase, which shares a highly conserved catalytic domain containing auto phosphorylating site [18]. It positively regulates cell cycle progression and plays a role in cell centrosome and spindle microtubules during mitosis [19]. On the other hand, it has been widely reported that *AURKA* is an oncogene to promote tumorigenesis in multiple types of cancer including solid tumors (such as bladder cancer [20,21], prostate cancer [22,23], colon cancer [24]) and hematological malignancies [18]. BUB1B also belongs to serine/threonine-protein kinase and could lead to cell death and slow growth in BC cells [25]. The abnormal regulation of *PRC1* contributed to cancer progress [26,27], such as prostate cancer and breast cancer [28,29]. But, the mechanism of *PRC1* in cancer is still unclear. *CCNB1* is a regulatory protein involved in mitosis and a critical cell cycle regulator of the G2/M checkpoint [30]. Previous studies have reported that *CCNB1* could participate in oncogene pathways among many kinds of cancers, such as BC and colorectal cancer [31–34]. And at initial stage of cancer, it was more recognized by the T cells [7,35]. Abnormal spindle-like microcephaly associated gene (*ASPM*) encodes a protein of 3477 amino acids with an NH2-terminal microtubule-binding domain and two calponin homology domains [36]. On the one hand, ASPM is a regulator of Wnt and stemness in pancreatic adenocarcinoma [37] which as a Wnt associated marker, it is not only could predict survival time but also could become a target therapy [38]. On the other hand, the function of *APSM* evidence has pointed out that it is an oncogene and its prognosis has been investigated in various cancers, such as epithelial ovarian cancer, gliomas, pancreatic and prostate cancer and liver cancer, as well as BC [37,39–43]. *MCAK* (also known as *KiF2C*), a member of the motor protein-13 motor family, is reported to undergo large conformational changes when it is converted from solution to microtubule-binding during its catalytic cycle [44]. The activity of *MCAK* is inhibited by Aurora B kinase through phosphorylation on multiple amino acids within its N-terminus [45,46]. Previous studies reported that the high expression of *KIF2C* could serve as an independent marker of poor prognosis in several tumors, including glioma, colorectal cancer, and gastric cancer [47–49], but the roles in BC reported less.

As for other hub genes, they were enriched in different GO BP terms. Targeting protein for Xenopus kinesin-like protein 2 (*TPX2*) is a microtubule-associated protein. The expression of *TPX2* is strictly regulated by the cell cycle. In general, *TPX2* exists in the G1 and S phases of the cell cycle and disappears at the end of cytokinesis [50]. A growing number of papers reported that the high expression of *TPX2* was connected with bad and shorter overall survival of patients in many tumors [51–53]. When using siRNA to knock down TPX2, the cycling-related proteins were down-regulated and cell apoptosis-related proteins were increased. It indicated that TPX2 is an important cell signaling molecular [52]. However, the mechanism of *TPX2* in BC is still unknown. *NUSAP1* (encodes nucleolar and spindle-associated protein 1), a nucleolar spindle-associated protein, has been reported that plays a complicated and sensitized role in cell division and mitotic progression, spindle formation, and stability controlled by phosphorylation [54]. *NUSAP1* was highly expressed in kinds of malignancies and correlated with poor prognosis in aggressive triple-negative BC. *TOP2A* is a DNA topoisomerase that participates in many processes during transcription and replication through altering DNA topological structure [55]. A significantly high expression level of *TOP2A* has been reported in many types of cancers [56–59]. And it was related to worse overall survival for various cancers [56,59]. Some researchers reported that *TOP2A* could induce apoptosis and suppress cell growth and invasion via AKT/ERK signaling pathways in colon cancer [7]. Kinesin family member 20A (*KIF20A*) is believed to modulate microtubule dynamics [60], which could promote the tumorigenesis and progression of prostate cancer and glioma [61], particularly the biochemical recurrence and metastasis [60,62]. Silencing *KIF20A* could induce prostate cancer cells to death and aberrantly activated *Gli2-KIF20A* axis which is crucial for the growth of hepatocellular carcinoma and predicts poor prognosis in hepatocellular carcinoma [63]. And KIF20A could induce paclitaxel resistance of BC [64]. Ribonucleotide reductase M2 subunit (*RRM2*), a rate-limiting enzyme involved in DNA synthesis and damage repair, plays important roles in many cellular processes such as cell growth, invasiveness, migration and senescence et al. [65]. *RRM2* as a tumor driver is frequently overexpressed in various malignancies [66–68]. Others found that the expression level of *RRM2* was correlated with invasion, cell differentiation, and metastasis in colorectal carcinoma [69], and correlated with lung cancer grade level [70]. Silencing *RRM2* attenuated melanoma growth, which was consistent with the maintenance of senescence-associated cell-cycle arrest [71]. Peroxisome proliferator-activated receptor gamma (*PPARG*) could induce cell cycle arrest, terminal differential, and anti-inflammatory [72,73], and induce G2/M cell cycle arrest by activating P38 in renal cancer and bladder cancer [74,75]. Furthermore, *PPARG* inducing the down-regulation of Wnt/beta-catenin pathway was observed and aberrant in many cancers [76]. Up-regulation of PPARG was correlated with downstream metabolic effectors. From a phenotypic point of view, this was associated with increased lung and lymph-nodes metastasis, indicating that the stratification method can be targeted for the treatment of aggressive diseases [77].

From the NCBI database, we could know that most of the hub genes were researched or verified, so we chose other non-hub genes and fewest reported three genes (*CDCA3, ZWINT, UBE2S*) to verified. These three genes were consistent with predicted results. CDCA3 is a FBOX protein, and is essential for cell mitosis [78], which take part in forming E3 ligase complex and relate to ubiquitination. It triggers cells to enter mitosis and dephosphorylation of CDC2 proteins in G2 phase. Some reports showed that Skp1-Cullin-F-box complex controlled the G1/S phase and led to cancers if became imbalance [79,80]. It had been found that *CDCA3* was significantly illustrated in the lung cancer samples and correlated with clinical progress [79]. And *CDCA3* was associated with worse RFS and OS in Luminal A breast cancer [80]. Some evidence also suggested that CDCA3 interacting with TRAF2 could activate NF-κB pathway in colorectal cancer. But, the mechanism of *CDCA3* in BC is still absent. *ZWINT* has same function with *CACA3*, could interact with E3 ligase and promote cell growth [81]. Reported studies suggested that *ZWINT* was highly expressed in various cancers and related with cancer progress, such as lung cancer [82] and liver cancer [83]. From our result, it may have the same function in BC. *UBE2S* is a member of the E2 family of ubiquitin-conjugating enzymes and collaborates with anaphase-promoting complex to prolong K11-linkages and polyubiquitin chains on substrates for 26 S proteasome-mediated degradation [84–86]. Also, it is responsible for Lys11-linkage ubiquitin modifications on β-catenin [87]. Past years have reported that *UBE2S* is preternaturally expressed in kinds of cancers and negatively relates with patients progress [88–90]. Highly expressed *UEB2S* facilitates cell proliferation and metastasis via targeting suppressor of Von Hippel-Lindau and p53 degradation [90]. Knocking down *UBE2S* could inhibit EMT cell signaling and inhibits invasion of cervical cancer [91]. But, the mechanism of *UBE2S* in BC is still poorly understood.

In conclusion, our study identified 13 hub genes and 3 non-hub genes (*AURKA, CCNB1, BUB1B, PRC1, TPX2, NUSAP1, TOP2A, KIF20A, KIF2C, RRM2, ASPM, PPARG*, non-hub genes are *CDCA3, ZWINT* and *UBE2S)* that might be involved in the progression of BC using multiple gene expression data sets and a series of comprehensive analyses of bioinformatics. These findings provide new insights into the diagnosis and treatment of the BC, while the main limitation of this research is lacking experiment to verify the hub genes expression level in the BC tissues and function in BC cells. Therefore, the further experimental studies are still needed to ensure our findings.

## HIGHLIGHT

(1) Identification of effective biomarkers of breast cancer.

(2)*AURKA, CCNB1, BUB1B, PRC1, TPX2, NUSAP1, TOP2A, KIF20A, KIF2C, RRM2, ASPM, PPARG*, and *CDCA3, ZWINT, UBE2S* may could as a breast cancer target genes.

(3) Cell division was mostly related.
